# The Significance of the Presence of Gilbert's Syndrome in Patients With Metabolic Dysfunction-Associated Steatotic Liver Disease (MASLD): A Retrospective Cohort Study

**DOI:** 10.7759/cureus.85074

**Published:** 2025-05-30

**Authors:** Arjuna P De Silva, Krishanni Prabagar, Anuradha S Wickramasinghage, Aruni A Wanniarachchi, Dileepa S Ediriweera, Madunil A Niriella, Hithanadura J De Silva

**Affiliations:** 1 Department of Medicine, University of Kelaniya, Ragama, LKA; 2 Department of Public Health, University of Kelaniya, Ragama, LKA

**Keywords:** cap score, gilbert’s syndrome (gs), liver fibrosis, liver stiffness measurement (lsm), metabolic dysfunction-associated steatotic liver disease (masld), transient elastography (vcte)

## Abstract

Introduction

Metabolic dysfunction-associated steatotic liver disease (MASLD) appears to be gaining increased attention as a potential global health issue. Closely associated with cardiometabolic risk factors, MASLD can progress to more advanced liver conditions, including fibrosis, cirrhosis, and hepatocellular carcinoma. It is characterized by the accumulation of lipids within hepatocytes, which leads to oxidative stress and hepatic inflammation. Gilbert’s syndrome (GS) is a benign hereditary condition marked by elevated levels of unconjugated bilirubin (UCB), a compound believed to possess antioxidant and anti-inflammatory properties. This study aims to explore the potential relationship between GS and MASLD, specifically investigating whether the presence of GS, and the consequent higher levels of UCB may influence the development or progression of liver fibrosis. It compares liver fibrosis and disease severity between MASLD patients with and without GS, using liver stiffness measurement (LSM), controlled attenuation parameter (CAP) score, Fibrosis-4 (FIB-4) index, and FibroScan-AST score (FAST score) as indicators of fibrosis and steatosis, with the goal of providing insight into the possible protective role of GS in the pathogenesis of MASLD.

Methods

This retrospective cohort study was conducted at Nawaloka Hospital, Sri Lanka, using medical records from 2022 to 2024. Data collected included anthropometric, biochemical, and sociodemographic parameters. Liver fibrosis was assessed using vibration-controlled transient elastography (VCTE) with the FibroScan® 502 device to measure liver stiffness. Metabolic dysfunction-associated steatohepatitis (MASH) with significant activity and fibrosis was assessed by FAST score. A power calculation was performed and a sample size of 189 participants was estimated to detect a mean difference in LSMs. Data analysis was performed using SPSS version 28 (IBM SPSS Statistics, Armonk, NY). Univariate analyses were conducted using the independent samples t-test and chi-square test, as appropriate.

Results

A total of 243 patients were initially screened, and following exclusion, 180 patients with MASLD were included, of whom 36 were identified as having GS (defined by total bilirubin >1 mg/dL with elevated UCB) and 144 without. No statistically significant difference in LSM was observed between participants with and without GS (P = 0.8919). GS was not significantly associated with the presence or severity of liver fibrosis. Similarly, GS showed no significant association with at-risk MASH (0.72). Increasing age (P = 0.0278), body mass index (BMI) (P = 0.0330), and blood sugar levels (P = 0.0257) were positively associated with liver fibrosis.

Conclusion

This retrospective cohort study, conducted at the largest private hospital in Sri Lanka, found no significant association between GS and the progression of MASLD, including the presence of MASH or the severity of hepatic fibrosis. Larger, prospective studies are needed to further investigate the potential role of GS in MASLD progression.

## Introduction

Metabolic dysfunction-associated steatotic liver disease (MASLD), formerly referred to as non-alcoholic fatty liver disease (NAFLD), is a liver disease that is associated with metabolic syndrome. It has emerged as a significant public health issue, with estimates suggesting that more than half of the global population could have MASLD by 2040 [1]. This condition is also strongly associated with cardiometabolic risk factors, including obesity, type 2 diabetes, and dyslipidemia, with approximately 30% of those diagnosed developing complications related to these underlying causes [2].

In addition to its impact on morbidity and mortality, MASLD imposes a substantial economic burden on healthcare systems worldwide [3]. Its pathophysiology is complex and multifactorial influenced by genetic predispositions, obesity, gut microbiota, and insulin resistance [4]. A defining feature of MASLD is the accumulation of lipids exceeding 5% within hepatocytes. These lipids, primarily fatty acids, are converted into triglycerides for storage. When present in excess, these fatty acids undergo oxidation in peroxisomes, leading to the generation of reactive oxygen species (ROS) that contribute to oxidative stress [5]. This oxidative stress plays an important role in driving hepatic inflammation, lipid peroxidation, and subsequent fibrotic changes [6]. Understanding the mechanisms behind lipid oxidation and inflammation has been a key focus of research in MASLD [7,8].

Similarly, Gilbert's syndrome (GS) is a common benign hereditary condition, affecting approximately 10-15% of the global population [9]. However, there is significant regional variation, with a prevalence of around 15% in Western populations and a notably higher prevalence in South Asian populations, particularly in countries like India, where carrier rates of relevant genetic variants have been reported as high as 32.8% [10,11]. It is characterized by mild and chronic elevations in unconjugated bilirubin (UCB) levels. This genetic condition is typically asymptomatic and underdiagnosed, with episodes often triggered by fasting, physical exertion, or dehydration. Recent research has highlighted the significance of its hallmark feature: elevated levels of UCB [12,13]. This molecule exhibits potent antioxidant and anti-inflammatory properties. These properties have been linked to reduced risks of oxidative stress-related conditions, including cardiovascular diseases, and have been associated with a reduced risk of diabetes, metabolic syndrome, obesity, and some autoimmune and neurodegenerative diseases [14,15].

It has even been shown to be protective against endometrial carcinoma and Hodgkin’s lymphoma, thereby producing lower mortality rates [16]. Since MASLD is part of metabolic syndrome, and given the important role of oxidative stress in MASLD progression, the antioxidant properties of bilirubin raise interesting questions about the potential influence of GS on MASLD outcomes. Could elevated bilirubin levels reduce hepatic oxidative damage and slow disease progression? Or might the altered bilirubin metabolism in GS exacerbate liver dysfunction under certain conditions? Furthermore, can the antioxidative effects of bilirubin improve prognosis in MASLD, potentially reducing the risk of hepatocellular carcinoma [17]? Despite the growing body of research in this area [18-20], the relationship between GS and MASLD remains poorly understood. Two studies conducted over a decade ago suggested a protective role of GS in NAFLD [21,22]. However, conflicting results in the existing literature highlight the need for further investigation [23].

This study aims to explore the relationship between GS and MASLD, with a particular focus on whether the antioxidant properties of bilirubin influence liver fibrosis and disease severity. The primary research question addressed is whether there are significant differences in liver fibrosis and steatosis between patients with and without GS. To evaluate this, the study compares liver stiffness measurements (LSM), controlled attenuation parameter (CAP) scores, and Fibrosis-4 (FIB-4) indices as markers of fibrosis, along with the FibroScan-AST (FAST) score as a marker for metabolic disease-associated steatohepatitis (MASH) activity, across the two groups. By examining these endpoints, the study seeks to provide insights into the potential protective role of GS in MASLD pathogenesis and to inform more novel strategies for disease risk stratification and management.

## Materials and methods

The study was conducted at Nawaloka Hospital, Colombo 02, the largest private healthcare institution in Sri Lanka, using clinical data from inpatients who received standard care between 2022 and 2024. The sample size was calculated based on prior literature [21,22] and power analysis, which estimated a total of 189 participants to detect a mean difference of 1.6 kPa in LSMs, assuming a pooled standard deviation of 3.68 kPa, 80% power, a two-sided alpha of 0.05, and an unequal group ratio. Informed written consent for the use of medical data was obtained prior to data collection. Participants were identified from medical records (Bed Head Tickets, BHTs) based on predefined inclusion and exclusion criteria, and eligible patients were included in the study cohort.

Data collection was carried out between December 2024 and March 2025 by a single trained reviewer using standardized methods to minimize inter-reviewer variability and observer bias. Participants with missing essential data were excluded to maintain dataset integrity. To reduce bias, rigorous inclusion and exclusion criteria were applied, and key clinical and metabolic parameters were assessed to control for known confounders. Although the retrospective design limited control over all potential sources of bias, multiple measures were implemented to enhance methodological rigor and validity.

Initially, 243 patients were identified through retrospective analysis; however, 63 patients were excluded due to missing data, resulting in a final study population of 180 patients. The study population included patients diagnosed with MASLD based on sonographic features. MASLD was defined by the presence of hepatic steatosis identified via transient elastography (FibroScan®), with a CAP score ≥248 dB/m, in conjunction with at least one metabolic dysfunction feature. These included type 2 diabetes mellitus, overweight/obesity (BMI ≥25 kg/m²), or other metabolic risk factors such as elevated fasting blood glucose (≥100 mg/dL), hypertension, or dyslipidemia, in accordance with current consensus criteria [24]. Patients were excluded if they had significant alcohol intake, viral hepatitis, other secondary causes of hepatic steatosis, or chronic liver disease of other etiologies such as autoimmune hepatitis or hepatocellular carcinoma. Additional exclusions included known causes of elevated bilirubin levels (e.g., hemolytic anemia), use of medications that affect bilirubin levels (e.g., oral contraceptives and corticosteroids), and pregnancy.

Potential confounding factors, including the use of statins, antidiabetic drugs, and hepatotoxic medications, were carefully considered. Participants with documented use of such medications were excluded or adjustments were made in the statistical analysis to account for their potential impact on liver fibrosis markers and liver enzymes.

Participants were then categorized into two groups based on their total bilirubin levels documented in past medical records. Those with total bilirubin levels greater than 1 mg/dL, predominantly unconjugated, and without any other identifiable cause of hyperbilirubinemia were classified as having GS and formed the test group. Relevant investigations, including complete blood counts, were reviewed to rule out hemolysis, and clinical history was assessed to exclude other underlying causes of hyperbilirubinemia. Participants with total bilirubin levels below 1 mg/dL were classified as not having GS and served as the control group.

Anthropometric, biochemical, and sociodemographic data were extracted. Liver fibrosis assessments, conducted as part of routine clinical care, were performed using the FibroScan® 502 device. Measurements included LS and CAP, both obtained by a single experienced operator who had conducted more than 1,000 prior scans to ensure consistency and reliability. In addition, the FIB-4 score and the FAST score were calculated.

Data analysis was performed using SPSS version 28 (IBM SPSS Statistics, Armonk, NY). Univariate analyses were conducted using independent t-tests, chi-square tests, and simple linear regression, as appropriate. A p-value of <0.05 was considered statistically significant.

Ethical approval for the study was obtained from the Ethics Review Committee of the Faculty of Medicine, University of Kelaniya. All data were extracted anonymously, and each participant was assigned a unique identification number to ensure confidentiality and prevent identification from the dataset.

## Results

Demographic and clinical characteristics of the participants are summarized in (Table 1).

**Table 1:** Demographic and Clinical Characteristics of the Participants Descriptive statistics are presented as the mean (standard deviation). Categorical data are presented as n (%), where n represents the number of participants, and the percentage refers to the proportion within each specific group. Given the sample sizes and the observed difference in liver stiffness, the posthoc power to detect this difference was estimated to be approximately 5%. CAP, controlled attenuation parameter; FAST, FibroScan-AST; BMI, body mass index; FBS, fasting blood sugar; FIB-4, Fibrosis-4

Variable	Gilbert's Syndrome(n=36)	Controls (n=144)
Age (years)	48.39 (9.73)	47.03 (10.85)
Gender	33 (91.67%/3 (8.33%)	115 (79.86%)/29 (20.14%)
BMI (kg/m²)	29.13 (5.37)	30.74 (5.94)
FBS (mg/dL)	115.97 (38.10)	114.79 (33.60)
HDL (mg/dL)	52.08 (15.43)	47.49 (12.22)
LDL (mg/dL)	125.22 (44.39)	119.47 (39.49)
Total cholesterol (mg/dL)	202.58 (47.83)	196.47 (42.53)
Total bilirubin (mg/dL)	1.50 (0.52)	0.58 (0.20)
CAP score (dB/m)	308.06 (41.38)	314.97 (38.16)
Liver stiffness (kPa)	10.53 (8.43)	10.33 (7.61)
FIB-4	1.28 (1.19)	1.07 (0.59)
FAST score	0.43 (0.27)	0.42 (0.22)

Evaluation of NIBM and serum bilirubin in participants with and without GS

**Figure 1:**
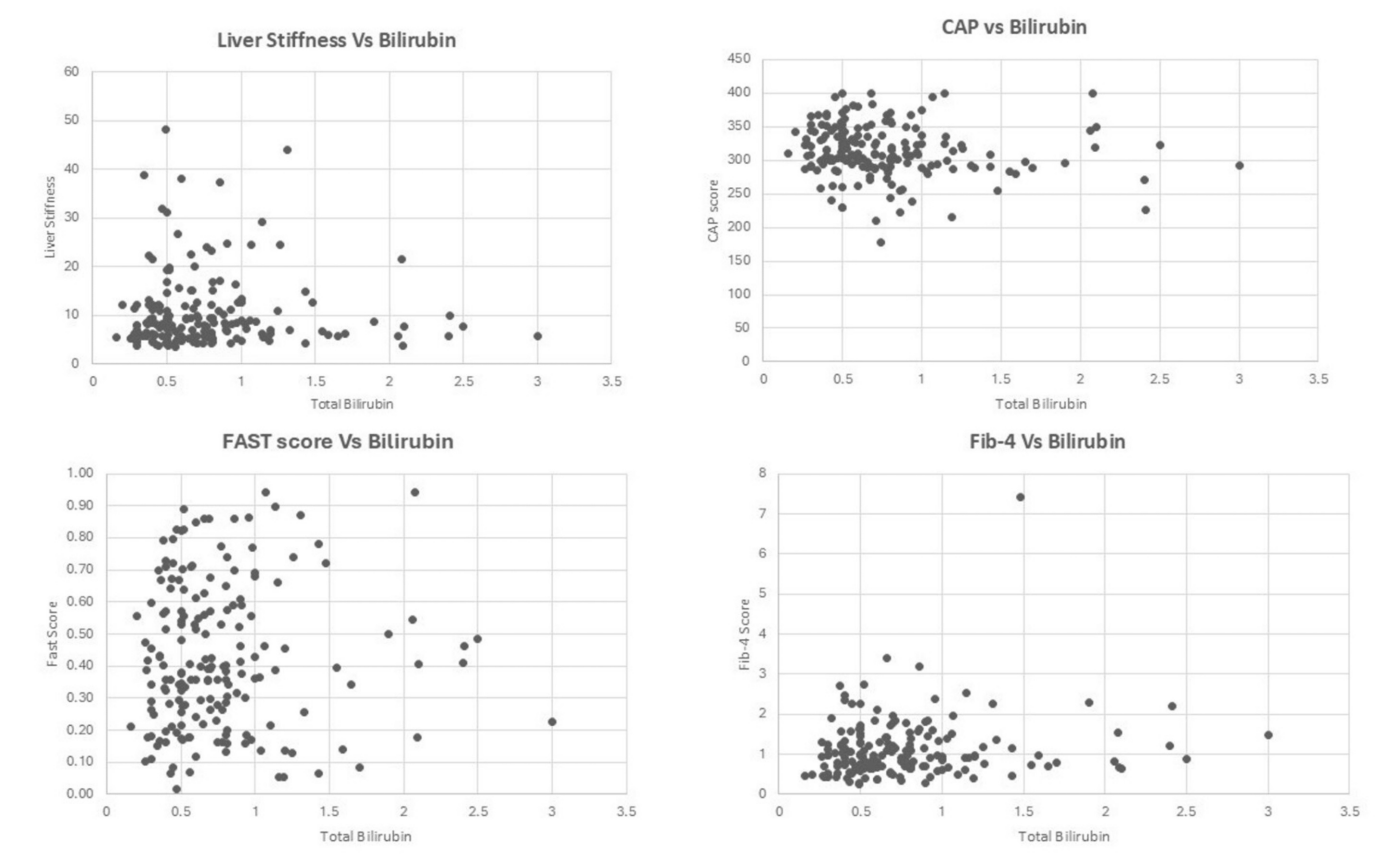
The Relationship Between Noninvasive Liver Biomarkers and Bilirubin Levels in Participants Liver stiffness is measured in kilopascals (kPa). The CAP score is measured in decibels per meter (dB/m). Serum bilirubin levels are measured in milligrams per deciliter (mg/dL). No significant correlation was observed between bilirubin levels and liver stiffness (P = 0.800), CAP score (P = 0.073), or FAST score (P = 0.709). A weak but statistically significant positive correlation was noted between bilirubin levels and FIB-4 score (P = 0.048). CAP, controlled attenuation parameter; FAST, FibroScan-AST; FIB-4, Fibrosis-4

The relationship between noninvasive liver biomarkers (NIBM) and bilirubin levels of participants was analyzed (Figure 1).

There was no significant correlation observed between liver stiffness and bilirubin levels (Pearson’s r = -0.019, df = 178, P= 0.800), CAP score and bilirubin levels (r = -0.135, df = 178, P= 0.073), or FAST score and bilirubin levels (r = 0.028, df = 178, P= 0.709). A weak but statistically significant positive correlation was found between FIB-4 score and bilirubin levels (r = 0.147, df = 178, P= 0.048), which may be influenced by the inclusion of age in the FIB-4 calculation, suggesting that age could act as a potential confounder in this association.

Following the correlation analysis, independent samples t-tests and Mann-Whitney U tests were performed to evaluate the association between GS and the NIBMs. The results are summarized in Table 2.

**Table 2:** Comparison of Noninvasive Liver Biomarkers Between Test and Control Groups *P-value of independent sample t-test **P-value of Mann-Whitney U test CAP, controlled attenuation parameter; FAST, FibroScan-AST; FIB-4, Fibrosis-4

Biomarker	Student’s T-test (t, df)	P-value*	Mann-Whitney U test (U)	P-value**
Liver stiffness	t = -0.136, df = 178	0.892	U = 2570.50	0.936
CAP score	t = -0.956, df = 178	0.340	U = 2120.00	0.091
FIB-4 score	t = -1.525, df = 178	0.129	U = 2422.50	0.549
FAST score	t = -0.219, df = 178	0.827	U = 2570.00	0.936

There were no statistically significant differences between participants with GS and the control group for liver stiffness (Student’s t-test, P = 0.892; Mann-Whitney U test, P = 0.936), CAP score (Student’s t-test, P = 0.340; Mann-Whitney U test, P = 0.091), FIB-4 score (Student’s t-test, P = 0.129; Mann-Whitney U test, P = 0.549), or FAST score (Student’s t-test, P = 0.827; Mann-Whitney U test, P = 0.936).

To further explore liver stiffness, values were categorized using established cut-offs of 8 kPa and 12 kPa, in accordance with the EASL-EASD-EASO clinical practice guidelines. These thresholds are recommended to help rule out (<8 kPa) or confirm (>12 kPa) the presence of advanced fibrosis [25]. The distribution of LSMs according to the cut-offs is summarized in Table 3.

**Table 3:** Distribution of Liver Stiffness According to Liver Stiffness Cut-Off Values Descriptive statistics are presented as n (%), where n represents the number of participants, and the percentage refers to the proportion within each specific group.

Variable	Gilbert's syndrome (n = 36)	Controls (n = 144)
Liver stiffness ≤8 kPa	20 (55.6%)	72 (50.0%)
Liver stiffness >8 kPa	16 (44.4%)	72 (50.0%)
Liver stiffness ≤12 kPa	27 (75.0%)	111 (77.1%)
Liver stiffness >12 kPa	9 (25.0%)	33 (22.9%)

A chi-square test was then performed to identify any significant relationship. The results are summarized in Table 4.

**Table 4:** Comparison of Liver Stiffness Based on Cut-off Values of 8 kPa and 12 kPa

	Test	Statistic	Df	P-value
Liver stiffness (cut-off: 8 Kpa)	Pearson’s chi-square test	χ² = 0.002	1	0.965
Liver stiffness (cut-off: 12 Kpa)	Pearson’s chi-square test	χ² = 0.168	1	0.682

There was no statistically significant association between GS and liver stiffness at either cut-off value (8 kPa: P = 0.965; 12 kPa: P = 0.682). Similarly, FAST scores were further evaluated using the recommended thresholds of 0.35 and 0.67 to categorize patients by risk. The distribution of FAST scores according to the cut-offs is summarized in Table 5.

**Table 5:** Distribution of FAST Scores According to Score Cut-off Values Descriptive statistics are presented as n (%), where n represents the number of participants, and the percentage refers to the proportion within each specific group. FAST, FibroScan-AST

Variable	Gilbert's syndrome (n = 36)	Controls (n = 144)
FAST score ≤0.35	12 (33.3%)	60 (41.7%)
FAST score >0.35	23 (66.7%)	84 (58.3%)
FAST score ≤0.67	27 (75.0%)	119 (82.6%)
FAST score >0.67	9 (25.0%)	25 (17.4%)

A chi-square test was then performed to identify any significant relationship. The results are summarized in Table 6.

**Table 6:** Comparison of FAST Scores Based on Cut-off Values of 0.35 and 0.67 FAST, FibroScan-AST

	Test	Statistic	Df	P-value
FAST score (cut-off 0.35)	Pearson’s chi-square test	χ² = 0.369	1	0.544
FAST score (cut-off 0.67)	Pearson’s chi-square test	χ² = 1.097	1	0.295

There was no statistically significant association between GS and FAST score at either cut-off value (0.35: P = 0.544; 0.67: P = 0.295).

Regression models

To further address the study’s objectives, specific hypotheses were developed and tested using simple linear regression models. The results have been summarized in Table 7.

**Table 7:** Simple Linear Regression Models of Variables on Noninvasive Liver Biomarkers CAP, controlled attenuation parameter; FAST, FibroScan-AST; BMI, body mass index; FBS, fasting blood sugar; FIB-4, Fibrosis-4; LSM, liver stiffness measurement

Predictor	Coefficients	Standard error	t-stat	P-value
LSM: Model 1				
Intercept	10.333	0.648	15.946	3.940
Gilbert's syndrome	0.197	1.449	0.136	0.892
Model 2: Age				
Intercept	4.711	2.616	1.800	0.074
Age	0.120	0.054	2.218	0.028
Model 3: BMI				
Intercept	3.965	3.037	1.306	0.193
BMI	0.211	0.098	2.149	0.033
Model 4: FBS				
Intercept	6.552	2.004	3.270	0.001
FBS	0.033	0.017	1.990	0.048
CAP score: Model 1				
Intercept	314.972	3.234	97.389	2.476
Gilbert's syndrome	-6.917	7.232	-0.956	0.340
Model 2: HDL				
Intercept	337.693	11.051	30.558	1.011
HDL	-0.498	0.220	-2.258	0.025
Model 3: FBS				
Intercept	292.048	9.997	29.215	8.044
FBS	0.187	0.083	2.249	0.026
FIB-4 score: Model 1				
Intercept	1.070	0.062	17.192	1.139
Gilbert's syndrome	0.212	0.139	1.525	0.129
FAST score: Model 1				
Intercept	0.424	0.019	22.468	7.528
Gilbert's syndrome	0.009	0.042	0.219	0.827
Model 2: Gender				
Intercept	0.319	0.039	8.168	5.590
Gender	0.130	0.043	3.018	0.003
Model 3: FBS				
Intercept	0.298	0.058	5.119	7.924
FBS	0.001	0.000	2.300	0.023

In the unadjusted regression analysis, GS was not significantly associated with liver stiffness (P = 0.892), CAP score (P = 0.340), FIB-4 score (P = 0.340), or FAST score (P = 0.340). In contrast, several metabolic and demographic factors showed significant associations.

Liver stiffness increased with age (0.12 kPa per year, P = 0.028), BMI (0.21 kPa per 1 kg/m², P = 0.033), and fasting blood sugar (FBS) (0.03 kPa per 1 mg/dL, P = 0.048). For the CAP score, higher HDL was associated with a reduction (-0.50 units per 1 mg/dL, P = 0.025), while higher FBS was linked to an increase (+0.19 units per 1 mg/dL, P = 0.026).

Regarding the FAST score, although GS had no significant effect, the male sex was associated with a 0.13 unit increase and FBS with a 0.001 unit rise per 1 mg/dL (P < 0.05 for both).

In summary, age, BMI, FBS, HDL, and gender emerged as key predictors of liver disease severity, while GS showed no independent association. These findings highlight the increased prevalence of fibrosis in older individuals and males and reinforce the importance of metabolic risk factor control in MASLD management.

## Discussion

The hypothesis for this study was based on two main factors. First, bilirubin's antioxidant properties are thought to play a critical role in reducing oxidative stress and inflammation, which are key drivers of MASLD pathophysiology, where oxidative stress in hepatocytes triggers progression to necroinflammation and fibrosis [4]. Second, GS has been shown to offer protection in a variety of conditions, including cardiovascular disease, ischemic stroke, lymphomas, autoimmune disorders, certain cancers, and NAFLD. Based on these, the study aimed to investigate whether the presence of GS is associated with differences in liver fibrosis and disease severity in patients with MASLD.

Two studies conducted over a decade ago suggested a protective role of GS in NAFLD [21,22], leading to the hypothesis that GS might similarly protect against the progression of MASLD. However, our study found no statistically significant difference between patients with GS and those in the control group. Several factors may account for this discrepancy. First, differences in study design and population characteristics may influence the observed effects. Many earlier studies were conducted in general populations or those with metabolic syndrome, whereas our cohort specifically comprised individuals with diagnosed MASLD, possibly representing a population with more advanced or established hepatic dysfunction. Second, it is possible that the protective effect of bilirubin is more pronounced in the early stages of metabolic dysfunction, with diminishing impact once steatotic liver disease is established. Finally, unmeasured confounders, such as genetic variability in UGT1A1 polymorphisms or differences in lifestyle and medication use, may have influenced outcomes and attenuated the expected protective association. Future prospective studies with genotyping and stratified analysis are warranted to clarify these complex interactions.

In our study, GS was diagnosed based on a serum bilirubin level greater than 1 mg/dL, predominantly unconjugated, while ruling out hemolysis and other causes of hyperbilirubinemia. This approach is consistent with widely accepted clinical diagnostic criteria, and it is generally agreed that genetic testing for UGT1A1 polymorphisms is not required for routine diagnosis [26].

The prevalence of GS in our cohort was 18.85%, which is similar to the 25.4% reported in a previous cohort from the region [21]. Globally, the prevalence of GS is approximately 10% [9]. It appears that patients with MASLD may have a higher prevalence of GS compared to the general population, although it is also possible that the elevation of UCB in MASLD is due to factors other than GS. The reasons for this increased UCB could be multifactorial. One possible explanation is the elevated level of heme oxygenase-1, which has been documented in MASLD patients [27]. This enzyme catalyzes the oxidative degradation of heme to biliverdin, which is subsequently reduced to bilirubin. Therefore, increased heme turnover in MASLD patients may contribute to the higher levels of UCB observed in this population.

We also observed a marked male preponderance in patients with GS (M:F = 11:1). This phenomenon is well documented globally and is presumed to be related to the effect of estrogen on the activity of UGT1A1 [28]. Estrogen is thought to upregulate the enzyme, thereby reducing UCB levels.

To the best of our knowledge, this is the first study to explore the relationship between GS and MASLD using transient elastography, the CAP score, and the FAST score. The advantage of using the FAST score is that it is a validated, non-invasive test for assessing MASH in our cohort [29]. As such, we evaluated liver fat content, the presence of MASH, and the degree of advanced fibrosis, which provided a clearer picture of the relationship between GS and MASLD. This study addresses a clinically relevant and underexplored area, the potential protective role of GS in patients with MASLD, adding novelty and value to the existing literature. The hypothesis is grounded in biologically plausible mechanisms, particularly the antioxidant and anti-inflammatory effects of UCB. The use of a retrospective cohort design is appropriate for this exploratory analysis and allows for efficient utilization of real-world clinical data. Such data enhance the practical applicability of the findings, particularly in understanding associations within routine healthcare settings.

However, our study has several limitations. First, its retrospective design introduces the potential for bias inherent in such analyses. Second, the study was underpowered to detect subtle differences in liver stiffness; the observed mean difference between the Gilbert’s and control groups was only 0.2 kPa with a pooled standard deviation of 7.78 kPa. Given the sample sizes (36 vs. 144) and a two-sided alpha of 0.05, the posthoc power to detect this difference was estimated to be very low (~5%), indicating limited ability to identify minimal effects. Additionally, liver fibrosis in our study was assessed using vibration-controlled transient elastography (VCTE) rather than liver biopsy. However, VCTE is now widely accepted in clinical guidelines as a reliable, non-invasive method for assessing liver fibrosis [25]. Another limitation is the reliance on single time-point laboratory values, which may not accurately reflect long-term bilirubin or metabolic profiles. The limited ethnic diversity of our study population also restricts the generalizability of the findings to broader, more heterogeneous populations. Moreover, the cross-sectional nature of the study limits our ability to draw conclusions about causality or disease progression over time. Clinical endpoints such as liver-related complications, fibrosis progression, or long-term patient outcomes were not assessed, leaving the impact of GS on the natural history of MASLD unresolved. These factors highlight the need for large-scale, longitudinal studies to better understand the clinical implications of GS in this context.

Despite these limitations, our study attempted to explore the relationship between GS and MASLD using multiple non-invasive measures that capture different aspects of liver health. Among our cohort, increasing age, higher BMI, and elevated blood glucose levels were identified as independent predictors of liver fibrosis, well-established risk factors that support the internal validity of our findings. Nevertheless, despite over a decade of research, the role of GS in MASLD remains inconclusive. One consistent observation is that GS does not appear to confer harm. Whether it offers protection, however, remains uncertain. A large-scale, prospective, multicenter study is ultimately needed to definitively answer this question.

## Conclusions

This retrospective cohort study, conducted at the largest private hospital in Sri Lanka with 180 participants diagnosed with MASLD, aimed to examine the relationship between GS and the progression of MASLD. The study found no significant association between GS and the presence of MASH or the degree of hepatic fibrosis in patients with MASLD.Future large-scale, prospective longitudinal cohort studies incorporating serial liver elastography assessments, advanced imaging modalities, and UGT1A1 genotyping are needed to better understand whether GS influences the progression of MASLD.
